# The Role of Chromatin Assembly Factors in Induced Mutagenesis at Low Levels of DNA Damage

**DOI:** 10.3390/genes14061242

**Published:** 2023-06-10

**Authors:** Tatiyana A. Evstyukhina, Elena A. Alekseeva, Vyacheslav T. Peshekhonov, Irina I. Skobeleva, Dmitriy V. Fedorov, Vladimir G. Korolev

**Affiliations:** 1 Chromatin and Repair Genetic Research Group of the Laboratory of Experimental Genetics, Department of Molecular and Radiation Biophysics, Petersburg Nuclear Physics Institute Named by B.P. Konstantinov of National Research Centre “Kurchatov Institute”, 188300 Gatchina, Russia; tat.evst@gmail.com (T.A.E.); pesh_vt@bk.ru (V.T.P.); skobeleva_ii@pnpi.nrcki.ru (I.I.S.); dfv85@mail.ru (D.V.F.); korolev_vg@pnpi.nrcki.ru (V.G.K.); 2 Laboratory of Molecular Genetic and Recombination Technologies, Kurchatov Genome Center—Petersburg Nuclear Physics Institute, 188300 Gatchina, Russia

**Keywords:** *Saccharomyces cerevisiae*, Asf1, NuB4 complex, Rad53, checkpoint

## Abstract

The problem of low-dose irradiation has been discussed in the scientific literature for several decades, but it is impossible to come to a generally accepted conclusion about the presence of any specific features of low-dose irradiation in contrast to acute irradiation. We were interested in the effect of low doses of UV radiation on the physiological processes, including repair processes in cells of the yeast *Saccharomyces cerevisiae*, in contrast to high doses of radiation. Cells utilize excision repair and DNA damage tolerance pathways without significant delay of the cell cycle to address low levels of DNA damage (such as spontaneous base lesions). For genotoxic agents, there is a dose threshold below which checkpoint activation is minimal despite the measurable activity of the DNA repair pathways. Here we report that at ultra-low levels of DNA damage, the role of the error-free branch of post-replicative repair in protection against induced mutagenesis is key. However, with an increase in the levels of DNA damage, the role of the error-free repair branch is rapidly decreasing. We demonstrate that with an increase in the amount of DNA damage from ultra-small to high, *asf1Δ*-specific mutagenesis decreases catastrophically. A similar dependence is observed for mutants of gene-encoding subunits of the NuB4 complex. Elevated levels of dNTPs caused by the inactivation of the *SML1* gene are responsible for high spontaneous reparative mutagenesis. The Rad53 kinase plays a key role in reparative UV mutagenesis at high doses, as well as in spontaneous repair mutagenesis at ultra-low DNA damage levels.

## 1. Introduction

The use of a signal transduction network by the DNA damage response is crucial to postponing cell cycle progression and facilitating the repair of damaged DNA [1]. Despite being aware that DNA damage checkpoints are essential to maintain genome integrity, there is still a crucial question of how the cell manages to balance between checkpoint arrest and cell proliferation in the presence of constant endogenous and exogenous sources of DNA damage [2]. Although DNA repair pathways show noticeable activity below a certain dose threshold for genotoxic agents, this threshold may vary depending on the organism, cell type, and the agent that causes damage [3].

Histone chaperones play a key role in regulating chromatin dynamics [4,5]. In yeast, Asf1 serves as a histone H3/H4 chaperone that is highly conserved among eukaryotes. It was discovered that it caused a loss of position-dependent transcriptional silencing when overexpressed, making it an essential component of chromatin-related functions [6]. Asf1 facilitates multiple processes, including the assembly and disassembly of nucleosomes, the cellular response to DNA damage, and the coordination of DNA-templated activities, such as replication, transcription, and repair [7,8,9]. The deletion of ASF1 renders cells sensitive to agents that induce DNA damage and replicational stress [8]. Asf1’s N-terminal core, which is highly conserved, is critical for its function and mediates its interactions with histones H3 and H4 [10,11], the chaperone complexes CAF-1 [8,12], and HIR (HIRA) [13].

Asf1 contributes directly to the replication-independent integration of H3 and H4 into nucleosome core particles with the participation of HIR proteins [14]. In addition, Asf1 has an impact on the post-translational modification of histones. Specifically, it boosts lysine acetyltransferase activity responsible for modifying Lys9 and Lys56 of newly synthesized H3 and stimulates the Set2-dependent trimethylation of H3K36 in chromatin [15,16,17,18,19,20]. Asf1 and Rad53 interact both physically and functionally. During normal yeast cell growth, Rad53 forms a stable complex with Asf1 which is released in response to both DNA damage and DNA replication arrest [21,22,23]. Upon genotoxic stress, Rad53 is activated through its phosphorylation on multiple sites via the action of the Mec1 and Tel1 kinases upstream, as well as by its own autophosphorylation [14,24,25,26].

Asf1 interacts with acetylated H3/H4 histones through a mechanism that is regulated by Rad53 and DNA damage response. In budding yeast lacking Asf1, the levels of certain DNA replication proteins, such as replication factor C (RFC), proliferating cell nuclear antigen (PCNA), and polymerase ε (Pol ε), decrease significantly at stalled replication forks [27].

The NuB4 complex has a close association with Asf1p, which controls the flow of histones into different chromatin assembly pathways. This bond has been recognized as evolutionarily conserved and has been observed in various organisms [20,21,28,29]. Hat1 is catalytic subunit of NuB4 complex that includes Hat1p, Hat2p, and Hif1p [30,31]. Hat2p possesses histone chaperone activity and is believed to facilitate the interactions of various NuB4 complexes with histones [31,32,33,34,35]. Hif1p is a member of the N1 family of histone chaperones and, specifically, binds to histones H3 and H4. Hif1p may play a role in depositing histones onto DNA, suggesting that Hat1p could be directly involved in the chromatin assembly process [30]. The NuB4 complex physically interacts with Hsm3p, which we previously identified as a functional subunit of the NuB4 complex [36,37,38,39]. Asf1p is linked to newly synthesized histones that bear the acetylation pattern characteristic of the Hat1p action [29,40,41,42]. Furthermore, a direct physical association between Asf1p and the NuB4 complex has been identified, implying that the NuB4 complex might directly transfer newly synthesized histones to Asf1p [43].

In this study, we focused on the mutational processes that occur during a local checkpoint. 

Our findings revealed that the error-free branch of post-replicative repair plays an important role in guarding against induced mutagenesis when the level of DNA damage is exceedingly low. However, when the levels of DNA damage escalate, the effectiveness of the error-free repair branch declines rapidly. The decrease in the mutagenesis efficiency is linked to the fluctuation in the activity of Rad53 kinase, the fluctuation in the RNR complex’s activity, and, therefore, improving the accuracy of the error-free branch of post-replicative repair.

The main idea of this study was to study the effect of low doses of ultraviolet radiation on biological processes, including repair processes, in *S. cerevisiae* yeast cells. Yeast housekeeping genes are known to be evolutionarily conservative and have a high degree of homology with mammalian/human genes. Therefore, the data obtained from the yeast model can be extrapolated to such a complex organism as the mammalian organism. Our study is of fundamental importance and will help to better define the biological effects of low doses of radiation at the cellular level. The importance of studying DNA repair processes and resistance to UV-induced DNA damage lies in the increased incidence, such as skin cancer, in patients with the genetic disease xeroderma pigmentosum [44,45], which is caused by the mutations in the genes responsible for nucleotide excision repair (NER) or the damage bypass by error-prone DNA polymerases. Four highly conserved DNA damage response mechanisms are known to contribute significantly to the response to ultraviolet radiation exposure: NER; damage tolerance *RAD6*; homologous recombination; and DNA damage checkpoint [46].

## 2. Materials and Methods

**Strains:***S. cerevisiae* strains used in this work are described in Table 1.

LMG-3031 (*MATα ade2Δ-248 ura3-160,188 leu2-3,112 trp1*) strain was transformed with those modules, and the transformants were selected on plates with selective media without uracil. The single *rad52Δ* (CAY-13), *hsm6-1* (6B-SVK-312), *pph3Δ* (9-DVF-3031), *hat1Δ* (CAY-2), *hsm3Δ* (5-LMG-3031), *hif1Δ* (CAY-3), and *rad30Δ* (4-EAA-3031) strains were previously described [36,46]. The double *asf1Δ rad52Δ* (DVF-17), *asf1Δ hat1Δ* (DVF-18), *asf1Δ hsm3Δ* (DVF-19), *asf1Δ hif1Δ* (DVF-21), *asf1Δ pph3Δ* (DVF-22), and *asf1Δ rad30Δ* (TAE-156) mutants were obtained by replacement of *ASF1* gene in *rad52Δ*, *pph3Δ*, *rad53-HA-F* (10-DVF-3031), *hat1Δ*, *hsm3Δ*, and *hif1Δ* strains, according to the same procedure. All mutants were PCR-verified.

**Media:** Standard yeast media of complete and minimal composition were used in this work [51]. In some experiments, a liquid YPD was used without the addition of agar. When working with auxotrophic mutants, metabolites required for growth were added to the minimal medium at a rate of 20 mg/l for amino acids and 3 mg/l for nitrogen bases. As a selective medium for accounting for the frequency of canavanine resistance mutations, a minimal medium was used with the addition of a liquid YPD in an amount of 10 mL/l and required for the growth of amino acids and nitrogenous bases. Depending on the strains used, canavanine concentrations were up to 80 mg/l. Taking into account the frequency of induced mutations at five loci, YPD with alcohol instead of glucose was used, the composition of which was described earlier [52].

**Sensitivity against UV-irradiation:** Cell-killing tests were performed on Petri dishes by overnight culturing the corresponding strain in liquid YPD at 30 °C. The cells were washed and resuspended in water at a density of 1 × 10^7^ cells/mL. Cells were irradiated with a BUV-30 UV lamp (UV-C range). Aliquots were taken at different times, diluted, and seeded on Petri dishes with YPD to determine the number of surviving cells.

**Mutation frequency:** Mutation tests were performed on Petri dishes by the overnight culture of the corresponding strain in liquid YPD at 30 °C. The cells were washed and resuspended in water at a density of 1 × 10^7^ cells/mL. The cells were irradiated with a BUF-30 UV lamp. Aliquots were taken at different times, diluted, and seeded on Petri dishes with YPD to determine the number of survivors. To determine the mutation frequency, undiluted aliquots were placed on a YPD medium with alcohol instead of glucose, the composition of which was described earlier [52].

**Mutation rates:** The mutation rate was determined according to the methods: fluctuation test [53] and ordered seeding [54]. The first method allows determining the rate of spontaneous mutations of yeast cells during rapid growth on a complete medium. After incubation for three days, 12 separate colonies were evaluated; each colony was suspended in 1 mL of water and seeded on a selective medium with canavanine in the concentration, excluding the possibility of growth of canavanine-sensitive cells. We diluted the suspensions and plated them on a complete medium when estimating the number of cells that were seeded. After incubation for three or four days, we counted the number of canavanine-resistant colonies and the total number of cells on the Petri dish. The frequency of spontaneous mutations was estimated using a special formula [53].

Using the ideal order method, we can register the frequency of spontaneous mutations occurring during slow growth on a selective medium containing low concentrations of canavanine, in which cells are grown for eight to ten divisions. The cells were incubated in 2 mL of complete liquid medium for 2 days, then 1 mL of the grown culture was diluted in 5 mL of water. A special 150-device replicator was injected into the suspension, and the drops were placed on Petri dishes with the selective medium. After a 14-day incubation, the number of colonies resistant to canavanine and the total number of cells grown in 150 spots was counted. The number of nonmutant cells that grew was determined after washing cells from individual prints that did not visualize canavanine-resistant colonies. The mutation rate was determined by dividing the number of canavanine-resistant colonies by the number of cells in all prints [55].

A total of 5 repetitions of the experiment are shown in the graphs and tables, and the average values with a 95% confidence interval are given.

**Real-time PCR:** For conducting, real-time PCR was used on a CFX96 RT-PCR detection system (Bio-Rad, Watford, UK). The reactions were carried out in 25 µL volumes consisting of a 10 µL 2,5-fold reaction mixture for RT-PCR in the presence of SYBR Green I dye and Rox reference dye (Syntol, Moscow, Russia), 13.8 µL water, 1.0 µL of cDNA, and 0.1 (2 mM) respective primers (primers for gene *RNR3*: For *RNR3* 5′-ACACCTTTCATGGTTTATAAG-3′ and Rev*RNR3* 5′-CGACGATTTCACAACATAA-3′; for gene *ACT1*: For*ACT1* 5′-GAAGGTCAAGATCATTGC-3′ and Rev*ACT1* 5′-GTTGGAAGGTAGTCAAAG-3′).

PCR-cycling conditions were as follows: 1 cycle of 5 min at 95 °C, followed by 39 cycles of 15 s at 95 °C and 20 s at 52 °C. Melting curve analysis was 5 s incremental increases of 1 °C from 55 °C to 95 °C.

Control reactions with primer and template-free reaction mixtures were included. Two biological and three technical replicates were performed for each sample. The results were processed using the CFX Manager program.

**Statistical analysis:** Experimental data are shown as the means, standard deviations from at least three biological replicates, and statistical differences were determined by the Student’s *t*-test. Significance was determined at the level of *p* < 0.05.

**Figure 1:**
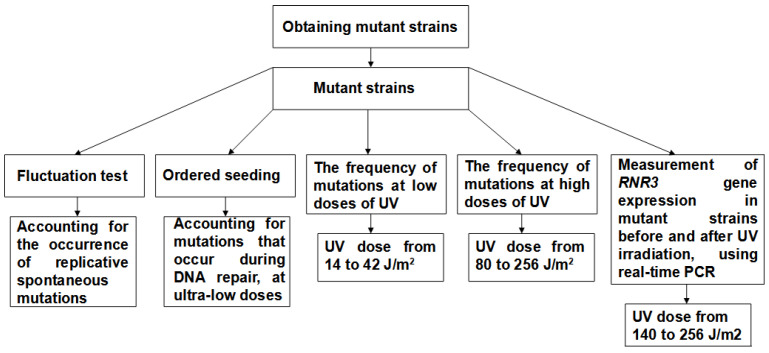
Flowchart summarizing the experimental part.

A flowchart summarizing the experimental part of this study is shown in Figure 1.

## 3. Results

Asf1 and CAF-1, H3-H4 histone chaperones in mammalian cells, exhibit a synergistic mechanism in restoring nucleosomes following nucleotide excision repair. [56]. To study the role of the NuB4 complex and Asf1p interaction in genetic processes, we studied the interaction of *asf1Δ* mutation with mutations in a number of genes that control various repair pathways and with mutations in genes encoding the subunits of the NuB4 complex.

**Effect of *asf1Δ* on spontaneous cell death:** Studies have demonstrated that the mutants for the *ASF1* gene affect the repair of double-strand breaks in yeast [57]. The double mutant *asf1Δ rad52Δ* differs from the parental single mutants in slow growth. To elucidate the reasons for this slowdown, we determined spontaneous cell death in the *asf1Δ* mutant and a number of its derivatives. The initial investigation involved evaluating the spontaneous mortality level of single *asf1Δ*, *rad52Δ*, and double *asf1Δ* and *rad52Δ* mutants in comparison to the wild-type strain. Lethal clones were identified by assessing how many clones had fewer than 16 cells after being cultured in a solid, complete medium for a day. Results revealed that both single mutants exhibited a distinct pattern and significantly altered the rate of spontaneous cell deaths when compared to the wild-type strain (Table 2). In the double *asf1Δ rad52Δ* mutants, we observed a synergistic interaction between *asf1Δ* and *rad52Δ* mutations. Thus, in a cell, the combination of *asf1Δ* mutation with *rad52Δ* mutation sharply decreases its viability and is the reason for the slow growth of double mutants. Taken together, these results suggest that Asf1 and Rad52 proteins play different roles in the spontaneous death of the yeast cells.

The interaction between the *asf1Δ* and *hsm3Δ* mutations was epistatic, which suggested the same pathway for the occurrence of lethal events disturbed by these mutations (Table 2).

**Spontaneous mutagenesis:** In order to determine the role of *asf1Δ* mutation in spontaneous mutagenesis, the Fluctuation test and Ordered seeding method were used to measure the levels of replicative and reparative spontaneous mutagenesis (Table 3). From Table 3, it can be seen that the presence of *asf1Δ* mutations in cells leads to a twofold increase in the rate of replicative mutagenesis. In this experiment, cells were grown on a rich medium, in which the time of one generation was as low as possible, which reduced the number of spontaneous DNA damage to a minimum. As a result, we observed a limited increase in the rate of spontaneous mutagenesis in *asf1Δ* mutant cells.

In the Ordered seeding test, the cells were grown on a selective medium, on which the time of one generation increased many times, which led to a sharp increase in the number of DNA damage during this period. Thus, in this test, we can measure the rate of spontaneous reparative mutagenesis under conditions of extremely low levels of DNA damage. As can be seen from Table 3, an increase in the generation time leads to a 25-fold increase in the rate of mutagenesis in the *asf1Δ* mutant.

Previous studies have shown that the genes *HAT1*, *HIF1,* and *HSM3*, encoding subunits of the NuB4 complex, participated in the control of replicative and reparative spontaneous mutagenesis [36,55,58]. In these experiments, *hat1Δ* mutation epistatized to *hsm3Δ* and *hif1Δ*. We measured the rate of spontaneous reparative mutagenesis in *asf1Δ hat1Δ, asf1Δ hsm3Δ,* and *asf1Δ hif1Δ* double mutants (Table 3). As you can see from this table, the interaction between *hat1Δ* and *asf1Δ* mutations is epistatic. At the same time, *asf1Δ* mutation epistatizes to *hsm3Δ* and *hif1Δ* mutations.

Interestingly, mutations in the gene-encoding subunits of the NuB4 complex and *ASF1* have virtually no effect on the rate of replicative spontaneous mutagenesis (Table 3). In cases when there were, nevertheless, noticeable increases in the rate of spontaneous mutagenesis measured by the Fluctuation test, this increase is explained by the large contribution of reparative mutagenesis to the total rate of spontaneous mutagenesis. On the other hand, the rate of reparative mutagenesis increased many times in all mutants. Thus, the low level of DNA damage in these mutants mediates high mutagenic efficiency.

At high doses of UV, *rad30Δ* mutation completely suppressed *hsm3*- and *hif1*-specific mutagenesis [36]. We assumed that a similar interaction would also be observed in the *asf1Δ rad30Δ* double mutant. To test this assumption, we deleted the *RAD30* gene in the wild-type strain and *asf1Δ* mutant. The *rad30Δ* single mutant showed spontaneous replication mutagenesis as the wild-type strain and high reparative spontaneous mutagenesis (Table 3). This result is consistent with the data obtained in experiments with low-dose UV irradiation of the *rad30Δ* mutant [56,59]. The authors showed that the main contribution to increased mutagenesis was made by Polζ when bypassing pyrimidine dimers. Spontaneous reparative mutagenesis in a double *asf1Δ rad30Δ* mutant was lower compared to a single *asf1Δ* mutant. If we take into account the contribution of Polζ to the level of spontaneous reparative mutagenesis in a single *asf1Δ*, which is not influenced by the Polζ, then the spontaneous mutagenesis level in the double *asf1Δ rad30Δ* mutant would be expected to decrease twofold. Based on these results, it can be assumed that the role of Polη polymerase in *asf1*-specific reparative mutagenesis of the major.

***rad53+HA-F* suppresses UV-induced *RNR3* activity:** Rad53 normally exists in a stable complex with Asf1 during the unperturbed yeast cell growth [21]. In the absence of Asf1, Rad53 is released from the complex and can be activated by autophosphorylation. Perhaps this activation plays an important role in asf1-specific mutagenesis. Previous research has indicated that specific phosphatases generate various types of Rad53 modifications necessary for an appropriate response to specific DNA damage [60]. Specifically, yeast cells employ phosphatase Pph3 to catalyze the dephosphorylation of the essential checkpoint kinase, Rad53 [61]. The deletion of the PPH3 gene results in increased Rad53 kinase activity. Pph3 forms a complex with the Psy2 subunit, which binds to kinase Rad53 and dephosphorylates it without the involvement of the third subunit. Another complex consisting of three subunits Pph3–Psy2–Psy4 dephosphorylates γH2A [62].

We hypothesized that disruption of the *PPH3* gene in the *asf1Δ* mutant would suppress *asf1*-dependent mutagenesis. The data shown in Table 3 confirm this assumption. The rate of spontaneous repair mutagenesis in the double mutant was lower than in any single mutant.

**Figure 2:**
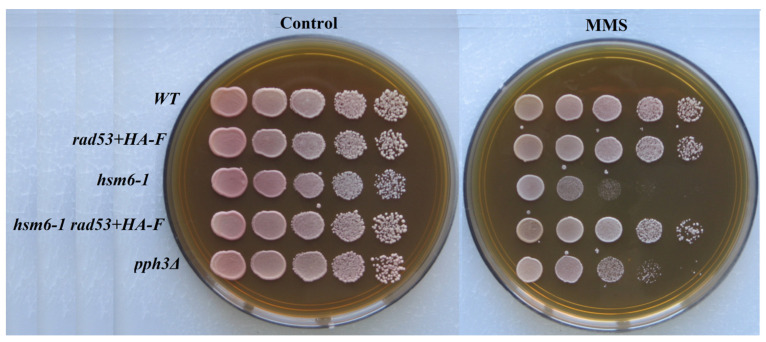
Sensitivity to MMS of wild-type (LMG-3031) strain and mutant strains *rad53+HA-F* (10-DVF-3031), *hsm6-1* (6B-SVK-312), *hsm6-1 rad53+HA-F* (DVF-23), *pph3Δ* (9-DVF-3031).

**Figure 3:**
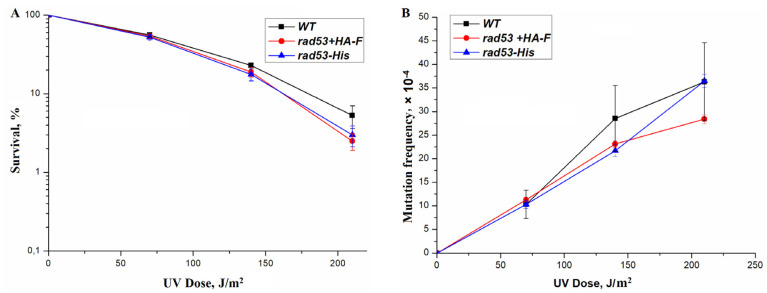
Sensitivity and mutagenesis in various mutant strains at different UV doses. Cells were irradiated at the indicated dose, the viable titer was determined, and the percentage of survivals was calculated. The mutation frequencies were determined as a ratio of the number of white colonies to the number of all colonies grown in a cup with complete medium. The mean ± SEM values obtained from 4 independent experiments are plotted. (**A**) UV-induced sensitivity in wild-type strain (LMG-3031) and mutant strains *rad53+HA-F* (10-DVF-3031) and *rad53-His* (25-DVF-3031); (**B**) UV-induced mutagenesis in wild-type strain (LMG-3031) and mutant strains *rad53+HA-F* (10-DVF-3031), *rad53-His* (25-DVF-3031).

Phosphorylated Rad9 interacts with the COOH-terminal fork head homology-associated (FHA) domain of Rad53. Inactivation of this domain abolished DNA damage-dependent Rad53 phosphorylation, G2/M cell cycle phase arrest, and an increase in *RNR3* transcription [63]. We hypothesized that a disruption of the structure of the C-terminal region of the protein might lead to the violation of some Rad53 functions. To test this assumption, we changed the C-terminal sequence of the Rad53 protein by adding the 6-His (6 histidines) and HA-F (influenza virus epitope hemagglutinin) sequences to the reading frame. These mutants were tested for resistance to MMS and UV-induced mutagenesis. Both mutants showed no difference from the wild-type strain in terms of UV resistance or UV-induced mutagenesis (Figure 2 and Figure 3).

Of importance, the MMS sensitivity of *pph3Δ* cells could be rescued by a hypomorphic allele of *RAD53* (*rad53-R605*) [64]. The *hsm6-1* mutation is an MMS-sensitive allele of the *PSY4* gene encoding a subunit of the PPH3 complex. We reasoned that if a *rad53+HA-F* is also a hypomorphic allele, it would rescue the MMS sensitivity of *hsm6-1* cells. Really, as can be seen from Figure 2, the MMS sensitivity of *hsm6-1* cells could be rescued by a *rad53+HA-F* allele. This result is consistent with our speculation that a violation of the structure of the C-terminal region of the protein may lead to deranging of some Rad53 functions (Figure 2).

**Figure 4:**
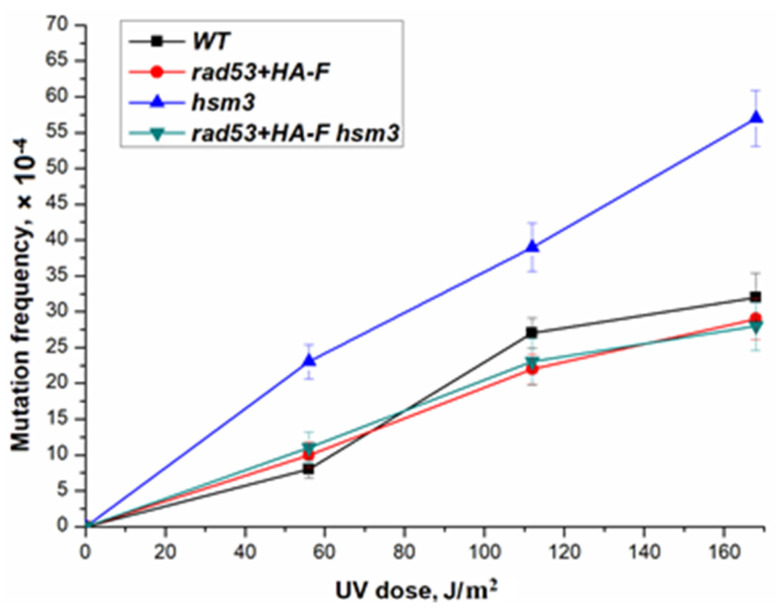
UV-induced mutagenesis in wild-type strain (LMG-3031) and mutant strains r*ad53+HA-F* (10- DVF-3031), *hsm3Δ* (5-LMG-3031), and *rad53+HA-F hsm3Δ* (DVF-24).

***rad53+HA-F* suppresses UV-induced mutagenesis of *asf1Δ*:** Next, we created the double mutant *rad53+HA-F hsm3Δ* and studied the effect of the mutant allele of the *RAD53* gene on the survival and mutagenesis of double mutant in UV irradiation. As can be seen from Figure 4, mutation of the *RAD53* gene epistatized to the *hsm3Δ* mutation, reducing the level of UV-induced mutagenesis to the level of a wild-type strain that is identical to the level of a single mutant *rad53+HA-F*. Therefore, mutations on the C-terminal domain of the *RAD53* gene suppress the mutator phenotype of the *hsm3Δ* mutant.

**Figure 5:**
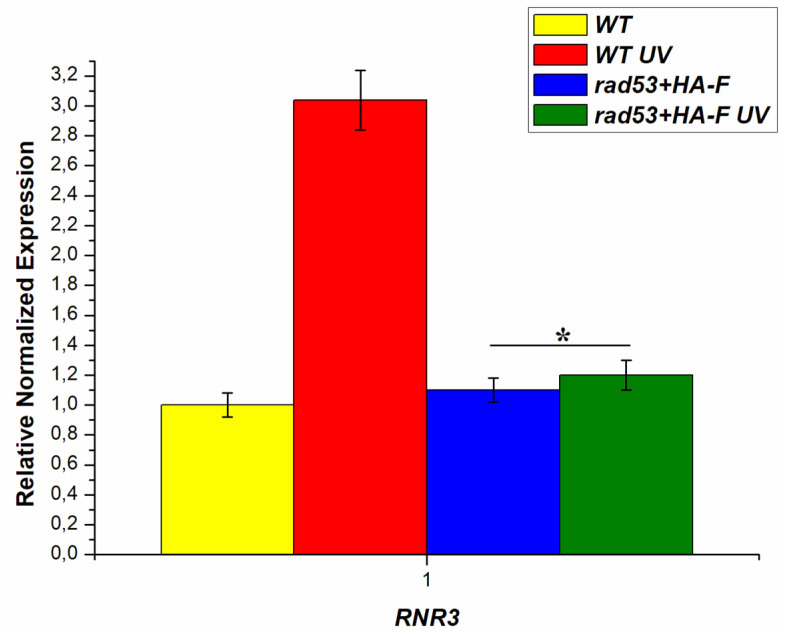
Relative normalized expression of the *RNR3* gene before and after irradiation with UV light of the corresponding strains (after UV irradiation, the cells were kept for two hours at 30 °C in a thermostat for induction), UV dose at 252 J/m^2^; * *p* < 0.05, Student’s *t*-test.

The mutator phenotype of the *hsm3Δ* mutant depends on the level of dNTPs; at low and high levels of dNTPs, *hsm3Δ*-dependent mutagenesis is suppressed. [36]. Due to this, we measured the expression level of the *RNR3* gene in *rad53+HA-F* mutant under normal conditions and after UV irradiation. Figure 5 shows that *rad53+HA-F* mutation does not increase the expression of the *RNR3* compared to the wild-type cells under normal growth conditions. After high-dose UV irradiation (252 J/m2), the expression of the *RNR3* gene increased three times, while in the cells of *rad53+HA-F* mutant, the level of expression of the *RNR3* gene did not change. These results are consistent with the data obtained in this work [63]. Next, we created a double mutant of *rad53+HA-F asf1Δ* and measured spontaneous mutagenesis in this mutant using two methods. The results of experiments with the double mutant showed that the non-inducible allele of the *RAD53* gene suppresses *asf1Δ* specific mutagenesis (Table 3). We then evaluated UV-induced mutagenesis in the wild-type strain and the double mutant at a low dose of 14 J/m^2^. The mutation rate was 5.7 × 10^−5^ and 4.8 × 10^−5^ for the wild-type strain and the double mutant, respectively. These results confirm that, as in the case of *him1Δ, hsm3Δ,* and *hif1Δ* mutants, *asf1Δ*-specific mutagenesis occurs only at intermediate levels of the *RNR3* gene expression. Taken together, these data show that at ultra-low levels of DNA damage, *asf1Δ* mutant exhibits extremely high reparative mutagenesis, which depends on the activity of Rad53 kinase.

Yeast cells do not orchestrate the transition to G1/S by generating dNTPs in the late G1 phase but rather use nucleotide shortage and fork arrest as physiological signal to activate dNTP synthesis [65]. We assumed that if the concentration of dNTPs in the G- and C-phases of the cell cycle is increased, replication will proceed without stops, and the checkpoint will not be activated. To test this assumption, we deleted the *SML1* gene and measured the level of spontaneous reparative mutagenesis in this mutant. As shown in Table 3, deletion of the *SML1* in wild-type cells causes a 25-fold increase in the rate of spontaneous repair mutagenesis compared to the wild-type strain. These data imply that elevated levels of dNTPs caused by the inactivation of the *SML1* gene are responsible for high reparative mutagenesis.

**Figure 6:**
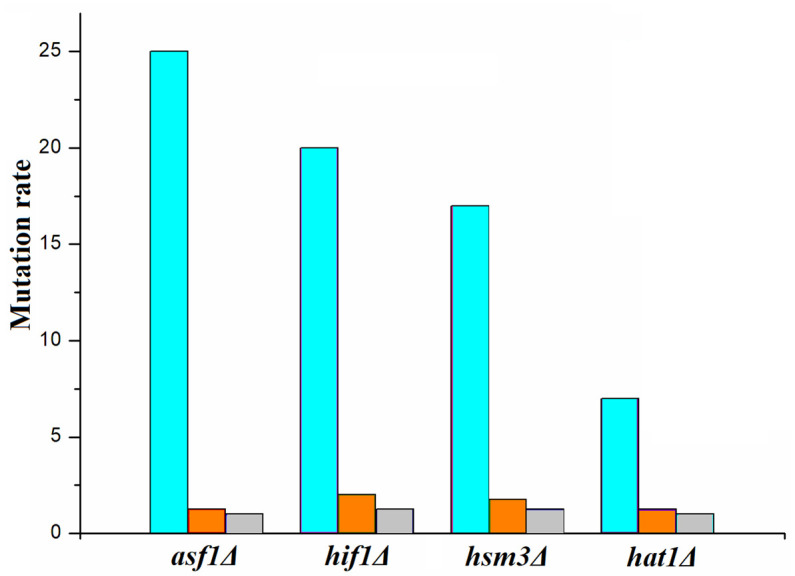
The degree of excess of the frequency of repair mutagenesis in various mutants over the wild-type strain at ultra-low (blue), low 10 J/m^2^ (orange), and high 180 J/m^2^ (gray) levels of DNA damage. The y-axis shows the ratio of the mutation rate in the mutant to the mutation rate in wild-type cells.

**The effect of low and high UV doses on strains with impaired reparative chromatin assembly:** Next, we measured the frequency of UV-induced mutagenesis at low doses (10 J/m^2^) in *asf1Δ, hsm3Δ, hif1Δ,* and *hat1Δ* mutants (Figure 6). As can be seen from Figure 6, the frequency of UV-induced mutagenesis in all mutants was higher than in the wild-type strain. However, unlike at ultra-low damage levels, low doses of UV caused only a modest increase in the frequency of mutagenesis in these mutants. This result led us to the idea of studying-induced mutagenesis at high UV doses. 

Previously, we showed that mutations in *HSM3* and *HIF1* gene-encoding auxiliary subunits of the NuB4 increase the efficiency of UV-induced mutagenesis at high doses. However, *hat1Δ* and *rad30Δ* mutants showed a level of UV-induced mutagenesis at high doses similar to the wild-type strain [36].

**Figure 7:**
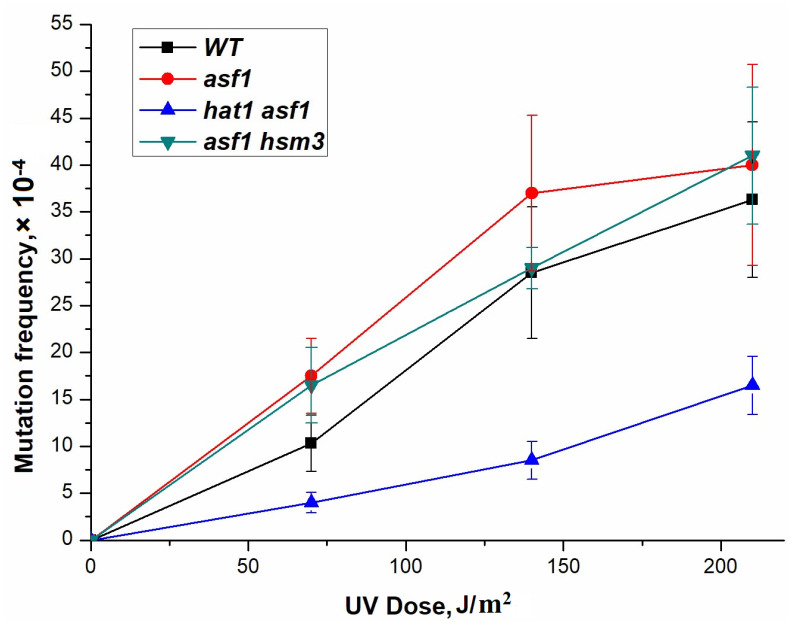
UV-induced mutagenesis in wild-type strain (LMG-3031) and mutant strains *asf1Δ* (4-DVF-3031), *hat1Δ asf1Δ* (DVF-18), and *asf1Δ hsm3Δ* (DVF-19).

To test the survival and mutagenesis of *asf1Δ* mutants, we performed experiments with UV light at high doses. We found that the *asf1Δ* mutant showed similar sensitivity to the wild-type strain (Figure 7). The frequency of occurrence of direct mutations in the *ADE4-ADE8* loci, induced by UV rays, was measured in a single *asf1Δ* mutant (Figure 7). As can be seen from this figure, *asf1Δ* mutation has no significant effect on UV-induced mutagenesis. The frequency of UV-induced mutations in the double *asf1Δ hat1Δ* mutant was lower than that of any single mutant (Figure 7). However, the interaction of *hsm3Δ* and *asf1Δ* mutations is of an epistatic character (Figure 7). In this case, the mutagenesis level of the double mutants is similar to a single *asf1Δ*.

Taken together, these results show that at high doses, the difference in UV-induced mutagenesis between the *asf1Δ* and *hat1Δ* mutants and the wild-type strain is eliminated, and both mutations epistate to *hsm3Δ* and *hif1Δ* mutations. Thus, with an increase in the amount of DNA damage from ultra-small to high, *asf1Δ*-specific mutagenesis decreases catastrophically (Figure 6). A similar dependence is observed for mutants for gene-encoding subunits of the NuB4 complex. All these mutations impair the quality of chromatin repair assembly and affect the level of dNTPs.

**Figure 8:**
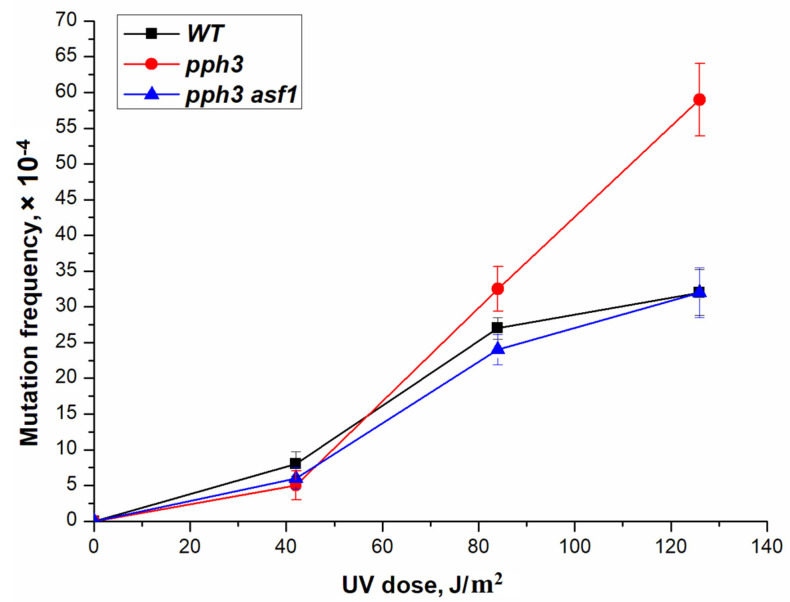
UV-induced mutagenesis in wild-type strain (LMG-3031) and mutant strains *pph3Δ* (9-DVF-3031) and *pph3Δ asf1Δ* (DVF-22).

Earlier, we indicated that the hyperphosphorylation of only the Pph3-specific sites of protein Rad53 and histone H2A increased the level of UV mutagenesis [49]. At high UV light doses, the frequencies of mutations in the *pph3Δ* mutant strain exceed the level of mutagenesis in the wild-type strain approximately twofold. This means that hyperphosphorylation of only the Pph3-specific sites of protein Rad53 and histone H2A increases the level of UV mutagenesis. We tested UV-induced mutagenesis at high doses in a double *asf1Δ pph3Δ* mutant. As with ultra-low levels of DNA damage, the asf1Δ *pph3Δ* double mutant had a lower mutation rate than any single mutant (Figure 8). Thus, the Rad53 kinase plays a key role in reparative UV mutagenesis at high doses, as well as at ultra-low doses.

## 4. Discussion

The canonical Mec1-Rad53 global checkpoint pathway does not function during normal DNA replication. Cells do not prepare for DNA replication by generating dNTP pools before entering S-phase but rather enter it with suboptimal dNTP levels [65]. The small level of deoxynucleotides upon entry into the S-phase is rapidly depleted; DNA polymerases are likely unable to efficiently utilize dNTPs at these low concentrations and must shut down and activate the Mec1-Rad53 pathway. The activation of the dNTP synthesis happens due to the accumulation of numerous single-strand gaps before the halted replication forks. Temporary activation of the Mec1-dependent pathway leads to dNTP synthesis via the degradation of Sml1 and activation of RNR, which is dependent on Rad53. Our findings suggest that cells do not initiate the transition to G1/S by producing dNTPs during the late G1 phase; instead, they employ the depletion of nucleotides and fork arrest as a biological cue to activate the dNTP synthesis. Upon entering the S-phase, replication forks will stop not only due to depletion of the nucleotide pool but also due to spontaneous or induced DNA damage. Thus, during replicative synthesis, additional gaps will accumulate on the damaged template of DNA, and the processes associated with replicative and repair synthesis will become closely integrated. With an artificially increased expression of RNR at the G1 stage, the replication of intact DNA will go on without stopping, and the activation of the replicative checkpoint will not occur. After the transit activation of Rad53 and the accumulation of a sufficient level of dNTPs, the replication forks on the intact template will be activated and quickly fill most of the single-strand gaps, which will lead to a rapid deactivation of Rad53. Replication forks that stop at the lesions will be the sites for the activation of post-replication repair and local activation of Rad53. Our data showed that rare gaps resulting from the arrest of replication polymerases on DNA damage generate mutational changes with high efficiency (Table 3).

In the present study, we examined the role of the Asf1 histone chaperone and various subunits of the NuB4 complex in spontaneous and induced mutagenesis at different levels of DNA damage. In this study, the results of spontaneous death in the *rad52Δ* and *asf1Δ* mutants show that recombinational repair under local checkpoint conditions plays a significant role in cell survival. It is possible that disturbances in the process of chromatin assembly caused by *asf1Δ* mutation have a predominant effect on the spontaneous death of *asf1Δ* cells. This conclusion is supported by the epistatic interaction of *asf1Δ* and *hsm3Δ* mutations. The observation that a *rad52Δ* mutation causes a synergistic increase in GCRs (gross chromosomal rearrangements) rates in *asf1Δ* mutant [66] is consistent with our data on the spontaneous death of the *rad52Δ asf1Δ* mutant (Table 2).

Hat1p and its associated factors are thought to act upstream of Asf1p as Asf1p has been found to be associated with newly synthesized histones that carry the acetylation pattern characteristic of Hat1p action [29,40,41,42]. In addition, a direct physical association between Asf1 and the NuB4 complex was recently reported suggesting the possibility that the NuB4 complex may directly transfer newly synthesized histones to Asf1p [43]. During the repair assembly of chromatin, the Asf1 protein forms a transit complex with the NuB4 complex. The analysis of spontaneous mutagenesis in the *asf1Δ hat1Δ* double mutant shows that Asf1-specific spontaneous repair mutagenesis is completely dependent on the normal functioning of the NuB4 complex and ultimately results from the activity of Rad53 kinase.

When the DNA is damaged, the checkpoint kinase Rad53 regulates the expression efficiency of the RNR complex. The absence of the Asf1 protein in the cell affects the activity of the Rad53 kinase in two different ways. First, under normal conditions, Asf1 directly interacts with the checkpoint kinase Rad53 in a manner regulated by the checkpoint activation [22,23]. Furthermore, *asf1Δ* cells display increased levels of Rad53 phosphorylation. Moreover, Asf1, together with the NuB4 acetylase complex, is involved in the reparative assembly of chromatin, and disruption of this process also affects the activity of Rad53 kinase [36]. In this regard, the level of deoxynucleotides in *asf1Δ* cells will be higher compared to wild-type cells, and DNA replication will not stop at the beginning of the S-period, or it will stop much later. On the other hand, stopping the replication machine at spontaneous damage will persist. These events will support the activation of the local checkpoint. We have previously shown that the intermediary activity of Rad53 during DNA repair synthesis stimulates the replacement of accurate replicative polymerases with a highly error-prone polymerase Polη, which leads to an increase in the mutation rate [36]. This increase was completely dependent on the function of Rad30. The data in Table 3 show that the inactivation of the *ASF1* gene indeed leads to a dramatic increase in reparative mutagenesis. However, the role of Polη in this increase is not a key one since the level of spontaneous reparative mutagenesis in the *asf1Δ rad30Δ* double mutant, although lower than in the single *asf1Δ*, remains at a high level. Possibly, under conditions of a local checkpoint and absence of Polη, the role of another TLS polymerase Polζ increases.

**Figure 9:**
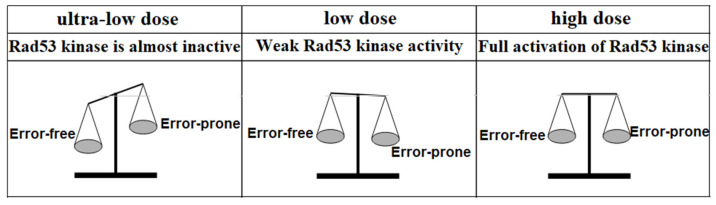
Relative contribution of the error-free branch of post-replicative repair to reparative mutagenesis at different levels of DNA damage.

In the present study, we examined the effect of the error-free branch of post-replicative repair on repair mutagenesis at various levels of DNA damage. The data obtained showed that at ultra-low levels of DNA damage, the role of the error-free branch of post-replicative repair in induced mutagenesis is key. However, with an increase in the levels of DNA damage, the role of this repair branch in induced mutagenesis is rapidly decreasing (Figure 6). This decrease in the efficiency of mutagenesis is associated with a change in the activity of the Rad53 kinase, the variability in the activity of the RNR complex, and improving the accuracy of the error-free branch of post-replicative repair (Figure 9).

## Figures and Tables

**Table 1:** Yeast strains used in this work.

Strains	Genotype	Origin
LMG-3031	*MATα ade2Δ-248 ura3-160, 188 leu2-3,112 trp1*	[47]
4-DVF-3031	*MATα ade2Δ-248 ura3-160,188 leu2-3,112 trp1 asf1Δ*	This study
CAY-13	*MATα ade2Δ-248 ura3-160,188 leu2-3,112 trp1 rad52Δ*	[48]
DVF-17	*MATα ade2Δ-248 ura3-160,188 leu2-3,112 trp1 asf1Δ rad52Δ*	This study
CAY-2	*MATα ade2Δ-248 ura3-160,188 leu2-3,112 trp1 hat1Δ*	[36]
DVF-18	*MATα ade2Δ-248 ura3-160,188 leu2-3,112 trp1 asf1Δ hat1Δ*	This study
5-LMG-3031	*MATα ade2Δ-248 ura3-160,188 leu2-3,112 trp1 hsm3Δ*	[48]
DVF-19	*MATα ade2Δ-248 ura3-160,188 leu2-3,112 trp1 asf1Δ hsm3Δ*	This study
CAY-3	*MATα ade2Δ-248 ura3-160,188 leu2-3,112 trp1 hif1Δ*	[36]
DVF-21	*MATα ade2Δ-248 ura3-160,188 leu2-3,112 trp1 asf1Δ hif1Δ*	This study
9-DVF-3031	*MATα ade2Δ-248 ura3-160,188 leu2-3,112 trp1 pph3Δ*	[49]
DVF-22	*MATα ade2Δ-248 ura3-160,188 leu2-3,112 trp1 asf1Δ pph3Δ*	This study
4-EAA-3031	*MATα ade2Δ-248 ura3-160,188 leu2-3,112 trp1 rad30Δ*	[50]
TAE-156	*MATα ade2Δ-248 ura3-160,188 leu2-3,112 trp1 asf1Δ rad30Δ*	This study
10-DVF-3031	*MATα ade2Δ-248 ura3-160,188 leu2-3,112 trp1 rad53+HA-F*	This study
25-DVF-3031	*MATα ade2Δ-248 ura3-160,188 leu2-3,112 trp1 rad53-His*	This study
6B-SVK-312	*MATα ade2Δ-248 ura3-160,188 leu2-3,112 trp1 hsm6-1*	[49]
DVF-23	*MATα ade2Δ-248 ura3-160,188 leu2-3,112 trp1 rad53+HA-F hsm6-1*	This study
DVF-24	*MATα ade2Δ-248 ura3-160,188 leu2-3,112 trp1 rad53+HA-F hsm3Δ*	This study
TAE-157	*MATα ade2Δ-248 ura3-160,188 leu2-3,112 trp1 rad53+HA-F asf1Δ*	This study
6-DVF-3031	*MATα ade2Δ-248 ura3-160,188 leu2-3,112 trp1 sml1Δ*	[50]

**Table 2:** Frequency of occurrence of spontaneous lethal clones.

Strain	Frequency
LMG-3031 (*WT*)	3.8 ± 1.4
4-DVF-3031 (*asf1Δ*)	9.3 ± 2.9
CAY-13 (*rad52Δ*)	10.1 ± 3.2
5-LMG-3031 (*hsm3Δ*)	5.0 ± 1.7
DVF-17 (*asf1Δ rad52Δ*)	33.6 ± 5.6
DVF-19 (*asf1Δ hsm3Δ*)	11.4 ± 2.55

**Table 3:** Spontaneous mutagenesis of resistance to canavanine.

Strain	Ordered Seeding, ×10^−7^	Fluctuation Test, ×10^−7^
LMG-3031 (*WT*)	3.0 ± 0.2	3.0 ± 0.2
9-DVF-3031 (*pph3Δ*)	52.7 ± 6.0	1.9 ± 3.4
5-LMG-3031 (*hsm3Δ*)	56.6 ± 3.4	3.6 ± 0.9
CAY-3 (*hif1Δ*)	52.1 ± 5.2	3.9 ± 0.6
CAY-2 (*hat1Δ*)	27.1 ± 4.1	4.1 ± 0.5
4-DVF-3031 (*asf1Δ*)	95.4 ± 10.4	7.4 ± 1.7
4-EAA-3031 (*rad30Δ*)	30.5 ± 4.1	4.7 ± 1.3
DVF-19 (*asf1Δ hsm3Δ*)	95.8 ± 6.1	12.5 ± 2.5
DVF-21 (*asf1Δ hif1Δ*)	87.3 ± 6.2	10.8 ± 2.7
DVF-18 (*asf1Δ hat1Δ*)	33.4 ± 3.6	5.8 ± 1.8
DVF-22 (*asf1Δ pph3Δ*)	19.3 ± 5.9	1.0 ± 0.6
TAE-156 (*asf1Δrad30Δ*)	75.1 ± 6.1	2.2 ± 0.2
6-DVF-3031 (*sml1Δ*)	74.8± 12.3	-

## Data Availability

No new data were created or analyzed in this study. Data sharing is not applicable to this article.
